# Simulation of catalase-dependent tolerance of microbial biofilm to hydrogen peroxide with a biofilm computer model

**DOI:** 10.1038/s41522-023-00426-z

**Published:** 2023-08-23

**Authors:** Philip S. Stewart, Mark Owkes

**Affiliations:** 1 Chemical & Biological Engineering, Montana State University, Bozeman, 59717 MT USA; 2 Mechanical & Industrial Engineering, Montana State University, Bozeman, 59717 MT USA

**Keywords:** Biofilms, Microbial ecology

## Abstract

Hydrogen peroxide (HP) is a common disinfectant and antiseptic. When applied to a biofilm, it may be expected that the top layer of the biofilm would be killed by HP, the HP would penetrate further, and eventually eradicate the entire biofilm. However, using the Biofilm.jl computer model, we demonstrate a mechanism by which the biofilm can persist, and even become thicker, in the indefinite treatment with an HP solution at concentrations that are lethal to planktonic microorganisms. This surprising result is found to be dependent on the neutralization of HP by dead biomass, which provides protection for living biomass deeper within the biofilm. Practically, to control a biofilm, this result leads to the concept of treating with an HP dose exceeding a critical threshold concentration rather than a sustained, lower-concentration treatment.

## Introduction

The ability of microorganisms in biofilms to withstand aggressive antimicrobial treatments has been long recognized^1–5^. The mechanisms behind this recalcitrance are multifaceted and may include delayed or incomplete diffusive penetration^6–8^, dormancy or persister cell formation^9–11^, protection by extracellular polymeric substances^12–15^, and active adaptive responses^16–20^. Here we conduct an in silico investigation of a particularly remarkable example of tolerance: the sustained persistence of viable biofilm during continuous exposure to a biocide at concentrations sufficient to rapidly kill free-floating cells.

Hydrogen peroxide (H_2_O_2_, HP) is a commonly used disinfectant and antiseptic. One of the beneficial features of HP is that it breaks down to innocuous end products, water and oxygen gas. In many microorganisms, this deactivation is catalyzed by enzymes known as catalases. Microorganisms deploy catalases to manage intracellular oxidative stress that is inherent to aerobic respiration^21–24^. Microbes often contain multiple genes coding for catalase enzymes. Some catalases can be induced by oxidative stress, while others are constitutively expressed. Catalases disproportionate HP to water and diatomic oxygen, completely neutralizing the antimicrobial power of the biocide. Concentrations of HP used to study oxidative stress responses in bacteria are commonly in the range of 10^−4^ to 1 mM^21–24^, whereas concentrations of HP used to treat biofilms are typically orders of magnitude larger (0.1 to 3% or 30 to 900 mM).

In a biofilm, consumption of HP by catalases in concert with the relatively slow process of molecular diffusion can lead to concentration gradients in HP with decreasing concentration with depth into the biofilm^25,26^. One might anticipate that as the top layer on the “front line” of the biofilm is killed, HP would penetrate further into the biofilm and eventually eradicate it completely. We used a computer model of biofilm accumulation to analyze this interaction and discovered a mechanism by which the biofilm can persist even in the face of prolonged treatment with a flowing solution of HP.

The one-dimensional mathematical model we used has a phenomenological structure that has a long-standing history in the biofilm literature^27–34^. It incorporates phenomena of microbial growth, consumption of a solute to support this growth, diffusion of the solute into the biofilm, detachment of microbial biomass from the topmost layer of the biofilm, killing of microbial cells by hydrogen peroxide, consumption of hydrogen peroxide by catalase-containing cells, and diffusion of hydrogen peroxide into the biofilm. The biofilm is simulated as a uniformly thick film growing in a continuous stirred-tank reactor. A companion paper describes the equations and the code for their numerical solution in detail^35^. We are not aware of any prior modeling work on the problem of continuous treatment of a biofilm with hydrogen peroxide.

## Results

### Biofilm thickness dynamics and sustained high viability during continuous dosing with hydrogen peroxide

**Fig. 1:**
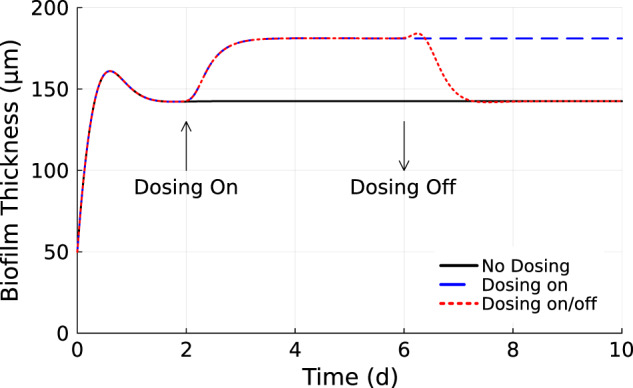
Biofilm thickness dynamics. Dosing off (solid black), no biocide treatment. Dosing on (dashed blue), biocide dosed continuously beginning at *t* = 2 days. Dosing on/off (dotted red), biocide dosed for 4 days beginning at *t* = 2 days, then removed to allow biofilm recovery.

Simulations of untreated biofilm followed a progression of biofilm thickness increasing rapidly from the initial thickness before plateauing at a stable steady-state value. This took about two days and the biofilm attained a steady state thickness of 140 microns (Fig. 1). Biofilm treated with 500 g/m^3^ HP became thicker in response to the biocide challenge, an unexpected outcome, then plateaued and stably withstood ongoing continuous dosing with the biocide (Fig. 1). When the biocide was discontinued, the biofilm returned within a day to its pre-treatment steady-state thickness (Fig. 1).

**Fig. 2:**
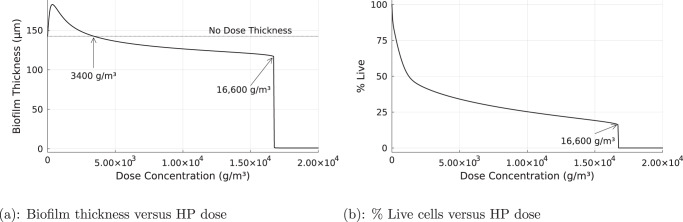
Impact of biocide concentration. Steady-state biofilm thickness versus biocide dose (**a**), and steady-state percentage live cells versus biocide dose (**b**). Continuous biocide treatment with HP concentrations ranging from 0 to 20,000 g/m^3^.

Biofilms persisted despite continuous exposure to high concentrations of HP. The steady state biofilm thickness was larger than the pre-treatment thickness for HP dose concentrations up to 3,400 g/m^3^ (Fig. 2(a)). Only when the HP concentration was increased beyond this amount did the steady-state biofilm thickness diminish below its pre-treatment value. Concentrations of HP greater than approximately 16,600 g/m^3^ eventually eradicated the biofilm (Fig. 2a) Dead cells were present in the biofilm after exposure to HP, but the percentage of live cells remained quite high even for large biocide dose concentrations. The log reduction in viable cells at steady state in response to continuous treatment with 16,600 g/m^3^ was 0.86.

### Concentration gradients of glucose and HP within the biofilm result in stratified metabolic and biocidal activity

**Fig. 3:**
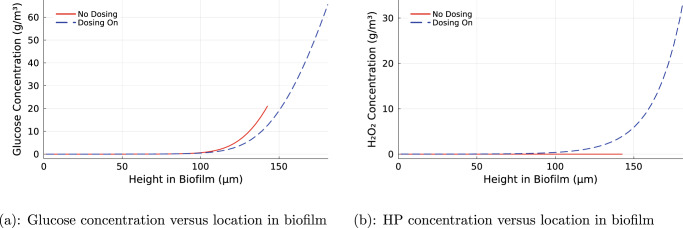
Glucose and H_2_O_2_ spatial variations. Steady-state glucose concentration (**a**) and H_2_O_2_ concentrations (**b**) within the biofilm. Results are presented without (red solid) and with (blue dashed) HP dosing.

Consumption of glucose by microorganisms in the biofilm resulted in the establishment of a concentration gradient of the growth solute within the biofilm (Fig. 3a). In the untreated (no hydrogen peroxide) case, glucose penetrated about 40 microns into the biofilm (Fig. 3a). When treated with hydrogen peroxide, the glucose concentration in the bulk fluid was higher, presumably because some of the microorganisms were killed and the overall consumption of the biocide was reduced. With the increased glucose concentration in the bulk fluid, glucose penetrated about 70 microns into the biofilm. In both cases, glucose was essentially depleted in the depths of the biofilm. In the untreated case, the region of nutrient availability constituted approximately 29% (40/140) of the biofilm whereas in the treated case the zone of glucose provision was roughly 39% (70/180) of the biofilm. HP also exhibited steep concentration gradients within the biofilm during exposure to the biocide (Fig. 3b). Thus, for both glucose and hydrogen peroxide there is a chemical stratification within the biofilm that creates regions of active growth and regions of biocidal activity. Both of these zones are skewed toward the top of the biofilm.

**Fig. 4:**
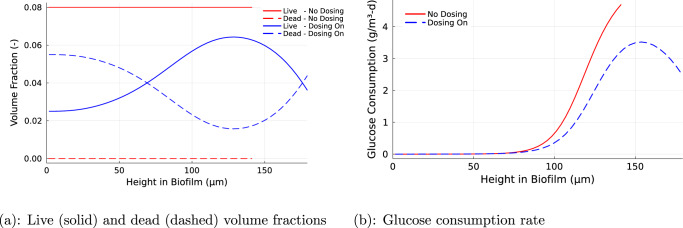
Biomass volume fractions and glucose consumption rate spatial variations. **a** Spatial distribution of biomass volume fractions of live (solid) and dead (dashed) biomass with no dosing (red) and with biocide dosing (blue). **b** Spatial distribution of glucose consumption rate within the biofilm with no dosing (red) and with biocide dosing (blue).

The distribution of live and dead cells within the HP-treated biofilm was complex and non-uniform (Fig. 4a). Live cells dominated in an interior stratum whereas dead cells dominated at the base of the biofilm and in a thin zone at the very top of the biofilm. This pattern reflects the location of the growing zone in the biofilm, which is also revealed by the peak in the pattern of glucose consumption (Fig. 4b). Dead cells are relatively concentrated at the top of the biofilm because this is where HP is at highest concentrations and disinfection is most rapid. Whereas live cells constituted 100% of cells in untreated biofilm, the mean live cell volume fraction in the treated biofilms was 56%. Note that the data in this plot requires additional time to achieve steady-state due to the slow dynamics near the bottom of the biofilm. Therefore, these results are from simulations with *t*_Final_ = 2000 days, which was determined to be sufficient after analyzing the results at a range of simulations with *t*_Final_ ∈ [10, 10000] days. The volumetric consumption rate of glucose inside the biofilm was reduced in HP treated biofilms (Fig. 4b). This was not due to the lower availability of glucose as the glucose concentration in the treated biofilm was higher than that in the untreated biofilm (Fig. 3b). The reduction in glucose consumption rate was likely due to the reduced volume fraction of live, metabolically active cells in the treated biofilm.

### Biofilm tolerance depends on dead cell neutralization of HP but not live cell neutralization of the biocide

**Fig. 5:**
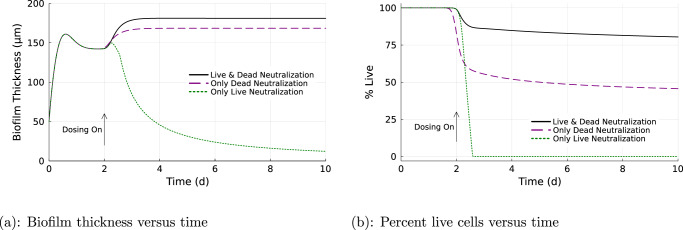
Biofilm protection depends on the hydrogen peroxide neutralizing activity of dead cells. Biofilm thickness versus time, (**a**), and percent live cells versus time, (**b**), with neutralization of hydrogen peroxide turned on for both live and dead cells (black solid), only dead cells (purple dashed), and only live cells (green dots).

**Fig. 6:**
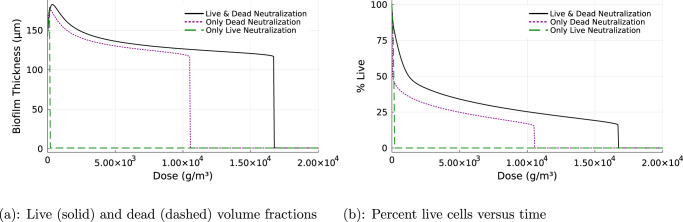
Impact of biocide concentration on biofilm thickness and percentage live cells. Effect on biofilm thickness (**a**) and percentage of live cells (**b**) when subjected to continuous biocide treatment with neutralization of hydrogen peroxide turned on for both live and dead cells (black solid), only dead cells (purple dashed), and only live cells (green).

When the neutralization of hydrogen peroxide mediated by dead cells was turned off, the biofilm became susceptible to killing (Fig. 5). Compared to the base case simulation that used both live and dead cell neutralization of HP, turning off dead cell neutralization resulted in the biofilm thickness decaying toward zero (Fig. 5a) as well as the elimination of live cells (Fig. 5b). Doses of HP, that in the baseline simulation were well tolerated, eliminated the biofilm in a simulation where dead cell neutralization of HP was turned off (Fig. 6). For example, whereas a biofilm in which both live and dead cells neutralize HP withstood a continuous dose of 16,600 g/m^3^, a dose of just 200 g/m^3^ fully decimated a biofilm in which dead cell neutralization was turned off (Fig. 6). Curiously, turning off the neutralization of hydrogen peroxide mediated by live cells had little influence on biofilm susceptibility (Figs. 5, 6). Therefore, it appears that the catalytic activity of dead cells versus HP is critical to robust biofilm protection, but not that of live cells.

To further analyze the impact of the neutralization of H_2_O_2_ by dead cells the biocide neutralization rate *k*_*B*:*D*_ was varied from 0 to 10 g_*H*_/m^3^/d and the thickness of the biofilm at steady-state was computed. At low neutralization rates of less than roughly 2 g_*H*_/m^3^/d the biofilm thickness was small indicating the dead cells were unable to neutralize the biocide fast enough to maintain the biofilm. However, for neutralization rates larger than roughly 4 g_*H*_/m^3^/d, the biofilm became thicker than the case without biocide treatment indicating the result is quite insensitive to the neutralization rate provided the rate is larger than a critical value.

### Biofilm tolerance to HP is modulated by the availability of glucose

**Fig. 7:**
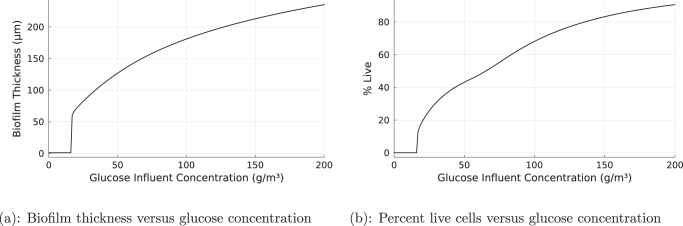
Biofilm tolerance of HP depends on glucose concentration. Steady-state biofilm thickness (**a**) and percent live cells (**b**) when subjected to continuous HP treatment at various glucose concentrations.

When the concentration of the growth-limiting solute, glucose, was decreased, the biofilm became less tolerant (Fig. 7). Decreasing glucose from 100 g/m^3^ in the base case to about 16 g/m^3^ resulted in the elimination of the biofilm when subjected to the standard HP dose (Fig. 7). Conversely, increasing the influent concentration of glucose made the biofilm somewhat more tolerant (Fig. 7).

## Discussion

A computer model of biofilm dynamics predicted robust protection of microorganisms in a biofilm from killing by hydrogen peroxide. In a base case simulation, the log reduction in viable cells after 1 day of continuous exposure of a biofilm to hydrogen peroxide at a mean bulk fluid concentration of 41.4 g/m^3^ over this interval was 0.063, corresponding to negligible killing. For comparison, if this concentration of HP was delivered to all of the cells in the biofilm for the same duration, the log reduction was calculated to be 9.0. The biofilm was protected because HP did not fully penetrate throughout the biofilm due to a reaction-diffusion interaction. Microbial cells in the outer regions of the biofilm consume and neutralize HP as it diffuses into the biofilm, leading to a standing gradient in biocide concentration within the biofilm (Fig. 3b).

The ability of aggregated microorganisms to neutralize hydrogen peroxide and prevent its penetration into the depths of a biofilm has been experimentally demonstrated. Hydrogen peroxide is biologically inactivated by enzymes, known as catalases, that convert the biocide to water and diatomic oxygen (O_2_). In a biofilm treated with sufficient HP, the evolution of gaseous oxygen can be visibly observed as effervescence emanating from the biofilm. Incomplete penetration of HP into biofilm was first experimentally demonstrated by Lu et al.^25^ using microelectrode technology. During 2 h of treatment with 3000 g/m^3^ of HP, gradients in the concentration of the biocide within the biofilm persisted. Stewart et al.^26^ used the same approach to demonstrate the failure of HP to penetrate to the bottom of a *P*. *aeruginosa* biofilm that was approximately 130 microns thick. In the same study, biofilms formed by a *P. aeruginosa katA* mutant that was deficient in catalase activity were fully penetrated by the same application of HP^26^. This result demonstrates that catalase activity alone provides sufficient reactive neutralization of hydrogen peroxide to prevent its effective penetration into a biofilm. Catalase activity has been shown to contribute to biofilm protection from HP in various microorganisms^16,36–40^.

**Fig. 8:**
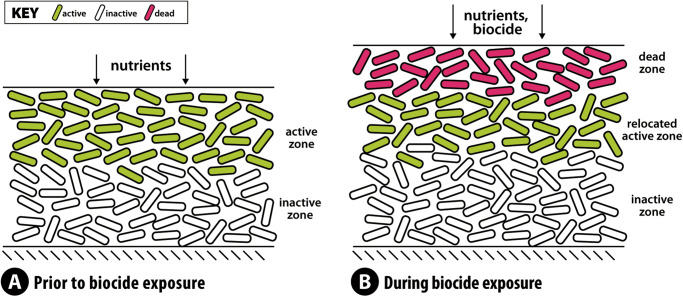
Conceptual model of sustained biofilm defense against continuous hydrogen peroxide exposure. **A** Prior to biocide treatment, a metabolically active zone (green) resides at the top of the biofilm with metabolically inactive cells in the interior of the biofilm (white). **B** During HP exposure, the top layer of the biofilm is composed mostly of dead cells (magenta). The metabolically active zone (green) relocates beneath the dead zone. Inactive cells (white) are located at the bottom of the biofilm. Growth of cells in the relocated active zone pushes a continuous supply of catalase-containing cells into the dead zone. Even though many of these cells die as they encounter HP in the upper layer of the biofilm, they retain their catalase activity and are able to consume HP and shield more deeply embedded cells from the biocide.

We propose a simplified conceptual model to explain the remarkable tolerance of biofilm to HP and the ability of the biofilm to maintain this defense indefinitely (Fig. 8). This model conceptualizes a zone at the top layer of the biofilm that is predominantly dead cells and an active zone beneath this layer that is predominantly living cells. In reality, the biofilm is not sharply stratified but harbors gradients in dead and live cells. The dead zone contains microbial cells, that while non-viable and unable to grow, nevertheless retain their catalase activity. Catalase does not require ATP or any regenerated co-factor and so this enzyme can continue to operate at full activity within a completely dead cell. The function of the cells in the outer dead layer is to provide a reactive shield that limits the penetration and access of HP to the viable cells in the interior of the biofilm. In the relocated active zone, viable bacteria continue to receive solute (glucose) and are able to grow. Indeed, because the biocide reduces viable bacteria at the surface of the biofilm, less glucose is consumed and the glucose concentration in the bulk fluid increases. This increases the concentration of glucose available to bacteria in the relocated active zone. The function of the growing cells in the relocated active zone is to provide a continuous supply of catalase-containing cells to replenish the cells in the dead zone. Dead cells at the very top of the biofilm detach and are released into the bulk fluid, but they are also continuously replaced by fresh cells coming from the growing layer beneath. There is an essential cohesive function of the biofilm matrix or extracellular polymeric substances in retaining dead cells and their associated catalase activity^37^. We hypothesize that the mechanistic features described above allow the biofilm to persist even in the face of long-term continuous exposure to HP.

The conceptual model outlined in the preceding paragraph is sufficient to provide enduring protection from HP. Phenomena that were not included in the computer model are therefore not essential to the prolonged tolerance we have simulated. The model did not incorporate the possibility of an adaptive response, i.e., induction of increased catalase activity upon exposure to HP. The ability of biofilm bacteria to induce catalase in response to oxidative stress is known^16,41^. If this effect were included in the computational model, one would expect that it would further enhance tolerance. However, it is not necessary to include an adaptive stress response to predict robust protection in the biofilm state. Similarly, the phenomena of dormancy or a shift in metabolic state that makes cells less vulnerable to HP was not included in our simulations. Because there are regions of the biofilm in which cellular metabolic activity is much reduced as a consequence of local deprivation for glucose, such a mechanism is plausible. If this effect were added to the computational model, it would also be expected to increase tolerance.

Most experimental data in the literature involve static, short-term challenge of biofilm with HP^15,42–47^. These are not appropriate comparisons for the simulations we have conducted that incorporate continuous provision of both growth-supporting nutrients as well as the biocide. Our predictions of increased biofilm thickness upon continuous exposure to moderate concentrations of hydrogen peroxide and complete elimination of biofilm at a distinct threshold high concentration have not been experimentally observed or tested. This represents an opportunity for future work by experimenters.

While the comparison to experimental results is therefore very limited, we examine two published studies that provide clues. In one study using *P. aeruginosa* biofilms grown in the drip-flow biofilm reactor, biofilms were exposed to a continuous flow of 50 mM HP (1700 g/m^3^) in a glucose-minimal medium for 1 h^16,26^ This resulted in removal of approximately 25% of the biofilm while viability of the remaining attached cells was reduced to approximately 80%. A quantitative comparison is not possible, but these effects are qualitatively consistent with our simulations (Fig. 2).

An interesting recent study, Zhang et al.^48^ used bacteria growing in microfluidic devices, interrogated via fluorescence microscopy, to investigate mechanisms of *Escherichia coli* biofilm response to treatment with HP under continuous cultivation. Biofilms treated with 300 g/m^3^ HP continued to grow and the *katG* catalase gene was expressed locally in a narrow region near, but interior, to the top of the biofilm. The HP caused oxidative stress in cells at the periphery of the biofilm as visualized by a reactive oxygen species sensitive fluorescent probe. During HP exposure, the zone of growth was located deeper within the biofilm. These observations are qualitatively consistent with the conceptual model diagrammed in Fig. 8.

The theory presented here should be applicable to any antimicrobial agent that can be enzymatically deactivated in a reaction that is not dependent on the viability of the cell. An example would be the class of *β*-lactam antibiotics^49^. These drugs, of which penicillin is a familiar example, contain a *β*-lactam ring that is cleaved by enzymes known as *β*-lactamases. This cleavage deactivates the antibiotic and is a primary mechanism of the spread of antibiotic resistance among pathogenic bacteria. In biofilms, *β*-lactamases have been shown to confer community protection^50–52^ Our model predicts that, just as for catalase-positive biofilms treated with HP, a biofilm formed by a *β*-lactamase-positive bacterium could exhibit sustained viability and even expansion when continuously treated with a penicillin-class antibiotic.

This work extends prior mathematical and computational models of biofilm inactivation. Previous reports have investigated the dynamics of antimicrobial penetration into biofilms^30,53^, the contribution of slow-growing or nutrient-limited microbes to antibiotic tolerance^29,32,54–56^, and the phenomenon of persister cells^34,55,57^. The current study makes a new contribution to understanding biofilm resilience by integrating the reaction-diffusion limited penetration of an antimicrobial with the continual regeneration of catalytic activity within a localized growing zone.

A practical prediction of this set of simulations is that the dose-response behavior of a biofilm treated with HP under continuous flow is highly non-linear. The biofilm will persist despite increasing the dose concentration well above concentrations that are lethal to planktonic microorganisms. This leads to the concept of a threshold treatment concentration that is needed to achieve biofilm control rather than a progressive dose response. Consider for example a biofouling control scenario for which biofilm thickness may be the most appropriate measure of success (rather than viability). HP dose concentrations up to 16,600 g/m^3^ only reduce the biofilm thickness from the untreated baseline by about 20%. Increasing the HP dose to 16,700 g/m^3^ results in the complete elimination of the biofilm and zero fouling. Thus, if HP is to be used in a biofouling control capacity, it would be important to establish the threshold concentration for biofilm elimination.

The results of this study also support the biofouling control strategy of reducing nutrients available to support biofilm growth. Reducing nutrient availability not only reduced innate fouling potential prior to biocide treatment, it also reduces the ability of the biofilm to persist during continuous HP exposure (Fig. 7).

## Methods

Simulations are conducted with Biofilm.jl^58^, which models a one-dimensional biofilm within a stirred tank reactor. Details are provided in^35^ on the governing equations, numerical methods, and application of the solver to a number of test cases. In this paper, the governing equations are described followed by a high-level description of the numerical methods.

The model solves for the concentrations of particulates and solutes within the tank (***X***_*t*_ and ***S***_*t*_) and biofilm (***X***_*b*_ and ***S***_*b*_) as well as the thickness of the biofilm (*L*_*f*_). For the biofilm studied in this paper, the particulates are live (*L*) and dead (*D*) biomass, and the particulates concentrations can be written as ***X***_*t*_ = [*X*_*t*:*L*_, *X*_*t*:*D*_] within the tank and ***X***_*b*_ = [*X*_*b*:*L*_, *X*_*b*:*D*_] within the biofilm. The solutes in this problem are glucose (*G*) and hydrogen peroxide (*H*), and the tank solute concentrations can be written as ***S***_*t*_ = [*S*_*t*:*G*_, *S*_*t*:*H*_] and the biofilm solute concentrations can be written as ***S***_*b*_ = [*S*_*b*:*G*_, *S*_*b*:*H*_].

### Tank liquid volume

The concentrations of particulates and solutes are solved for within the tank using

1 $$\frac{d{X}_{t:j}}{dt}=-\frac{Q}{V}\left({X}_{t:j}\right)+{J}_{\det :j}\frac{A}{V}+{R}_{X:j}({\boldsymbol{X}}_{t},{\boldsymbol{S}}_{t})$$

2 $$\frac{d{S}_{t:k}}{dt}=\frac{Q}{V}\left({S}_{{\mathrm{in}}:{k}}-{S}_{t:k}\right)+{J}_{{{{\rm{dif}}}}:k}\frac{A}{V}+{R}_{S:k}({\boldsymbol{X}}_{t},{\boldsymbol{S}}_{t})$$

for *j* ∈ {*L*, *D*} and *k* ∈ {*G*, *H*} with initial conditions

$${\left.{X}_{t:j}\right\vert }_{t = 0}={X}_{t:j}^{0}\quad \,{{\mbox{and}}}\,\quad {\left.{S}_{t:k}\right\vert }_{t = 0}={S}_{t:k}^{0}.$$

The first term on the right-hand-side (RHS) of Eqns. (1) and (2) describes the flow at a flow rate *Q* into and out of the tank with volume *V*. The flow of hydrogen peroxide into the tank controls the dosing and is given as

3 $${S}_{{{{\rm{in}}}}:H}(t)=\left\{\begin{array}{ll}0.0\quad &\,{{\mbox{for}}}\,t \,< \,{{{{\rm{time}}}}}_{1}\\ {{{{\rm{dose}}}}}_{1}\quad &\,{{\mbox{for}}}\,{{{{\rm{time}}}}}_{1}\le t\le {{{{\rm{time}}}}}_{2}\\ {{{{\rm{dose}}}}}_{2}\quad &\,{{\mbox{for}}}\,{{{{\rm{time}}}}}_{2}\, < \,t\end{array}\right.$$

The second terms on the RHS of Eqns (1) and (2) are the flux from the biofilm with surface area *A*. The flux of particulates is due to detachment from the top of the biofilm ${J}_{\det :j}={v}_{\det }{\left.{X}_{b:j}\right\vert }_{z = {L}_{f}}$ where ${v}_{\det }$ is the detachment velocity that is modeled with

4 $${v}_{\det }={K}_{\det }{L}_{f}^{2}$$

where ${K}_{\det }$ is the detachment coefficient. Particulate attachment is neglected as this process is expected to be important in the early stages of biofilm initiation but not in the long-term persistence of mature biofilms as is the focus in this investigation. The flux of solutes is due to diffusion between the tank and biofilm and is defined below in Eq. (12).

The third term is the rates defined for particulates as

5 $${R}_{X:L}({\boldsymbol{X}},{\boldsymbol{S}})={\mu }_{\max }\frac{{S}_{G}}{{K}_{M}+{S}_{G}}{X}_{L}-{k}_{{{{\rm{dis}}}}}{S}_{H}{X}_{L}\,\,{{\mbox{and}}}\,$$

6 $${R}_{X:D}({\boldsymbol{X}},{\boldsymbol{S}})={k}_{{{{\rm{dis}}}}}{S}_{H}{X}_{L}$$

which describes 1) the growth of live biomass using the Monod equation^59^ with maximum growth rate ${\mu }_{\max }$ and half-saturation constant *K*_*M*_ and 2) the death of live biomass due to the presence of hydrogen peroxide with *k*_dis_ being the disinfection neutralization rate coefficient. The rates for solutes are

7 $${R}_{S:G}({\boldsymbol{X}},{\boldsymbol{S}})=-\frac{1}{{Y}_{GL}}{\mu }_{\max }\frac{{S}_{G}}{{K}_{M}+{S}_{G}}{X}_{L}$$

8 $${R}_{S:H}({\boldsymbol{X}},{\boldsymbol{S}})=-{k}_{B:L}{S}_{H}{X}_{L}-{k}_{B:D}{S}_{H}{X}_{D}$$

which describes the consumption of glucose due to the growth of biomass with the yield coefficient of glucose on biomass *Y*_*GL*_ and the neutralization of hydrogen peroxide due to live and dead biomass with neutralization rate coefficients of *k*_*B*:*L*_ and *k*_*B*:*D*_, respectively.

### Biofilm volume

The concentration of particulates and solutes are solved for as a function of time *t* and location *z* within the biofilm using

9 $$\frac{\partial {X}_{b:j}}{\partial t}=-\frac{\partial v(z){X}_{b:j}}{\partial z}+{R}_{X:j}({\boldsymbol{X}}_{b},{\boldsymbol{S}}_{b})$$

10 $$\frac{\partial {S}_{b:k}}{\partial t}={D}_{b:k}\frac{{\partial }^{2}{S}_{b:k}}{\partial {z}^{2}}+{R}_{S:k}({\boldsymbol{X}}_{b},{\boldsymbol{S}}_{b})$$

for *j* ∈ {*L*, *D*} and *k* ∈ {*G*, *H*} with initial conditions

$${\left.{X}_{b:j}\right\vert }_{t = 0}={X}_{b:j}^{0}\quad \,{{\mbox{and}}}\,\quad {\left.{S}_{b:j}\right\vert }_{t = 0}={S}_{b:j}^{0}.$$

and boundary conditions

11 $${\left.\frac{\partial {X}_{b:j}}{\partial z}\right\vert }_{t,z = 0}=0$$

12 $${\left.\frac{\partial {S}_{b:k}}{\partial z}\right\vert }_{t,z = 0}=0\quad \,{{\mbox{and}}}\,\quad {\left.{D}_{b:k}\frac{\partial {S}_{b:k}}{\partial z}\right\vert }_{t,z = {L}_{f}}={D}_{t:k}\frac{{S}_{t:k}-{\left.{S}_{b:k}\right\vert }_{t,z = {L}_{f}}}{{L}_{L}}\equiv -{J}_{{{{\rm{dif}}}}:k}$$

Note that the particulate concentration is related to the volume fraction *P*_*b*:*j*_ by *P*_*b*:*j*_ = *X*_*b*:*j*_/*ρ*_*j*_ The first term on the RHS of Eq. (9) describes the vertical transport of particulates due to changes at deeper depths in the biofilm characterized by the growth velocity *v*(*z*), i.e.,

13 $$v(z)=\int\nolimits_{{z}^{{\prime} } = 0}^{z}\frac{1}{{P}_{{{{\rm{tot}}}}}}\mathop{\sum}\limits_{j=[L,D]}\frac{{R}_{X:j}({\boldsymbol{X}}_{b},{\boldsymbol{S}}_{b})}{{\rho }_{j}}\,d{z}^{{\prime} }$$

where ${P}_{{{{\rm{tot}}}}}=\mathop{\sum }\nolimits_{j = 1}^{{N}_{x}}{X}_{b:j}/{\rho }_{j}$ is the total volume fraction of particulates and *ρ*_*j*_ is the density of the particulates. The first term on the RHS of Eq. (10) is the diffusion of solutes through the biofilm with a diffusion coefficient *D*_*b*:*k*_. The rate terms, *R*_*X*:*j*_ and *R*_*S*:*k*_ use the expressions in Eqns. (5) to (8).

The boundary conditions at *z* = 0, the bottom of the biofilm on the solid surface, enforce a zero-flux condition. The diffusion flux boundary condition at the top of the biofilm (*z* = *L*_*f*_) defines *J*_dif:*k*_ and enforces conservation of mass at the surface of the biofilm, i.e., the flux into the biofilm (left-term) matches the flux through a mass transfer boundary layer within the tank on the surface of the biofilm of thickness *L*_*L*_ with a diffusion coefficient of *D*_*t*:*k*_ (middle-term).

### Biofilm thickness

The thickness of the biofilm is described by

14 $$\frac{d{L}_{f}}{dt}=v({L}_{f})-{v}_{\det }$$

with initial condition

$${\left.{L}_{f}\right\vert }_{t = 0}={L}_{f}^{0}.$$

The thickness depends on the growth velocity at the top of the biofilm (see Eq. (13)) and the detachment velocity (see Eq. (4)).

### Numerical methods

The equations are solved using Biofilm.jl which is written in Julia. The main workhorse of Biofilm.jl is the differential equation solver that is part of the DifferentialEquations.jl library^60^. To solve the equations, the partial differential equations (Eqs. (9) and (10)) are discretized on a one-dimensional grid within the biofilm with *N*_*z*_ = 50 grid cells and solved from *t* = 0 to *t*_Final_ = 100 days. Additional details on how the equations are discretized and solved are provided in Owkes et al.^35^. The source code used to run the simulations and produce the results is available at https://github.com/markowkes/Biofilm.jl/blob/HydrogenPeroxidePaper/HydrogenPeroxideDosing.jl.

### Simulation cases

The solver was run with a number of different cases. The standard parameters are provided in Table 1, which describes the dosing off case. Parameters for system geometry (V, A, Q) were arbitrarily chosen while values of diffusion coefficients^61,62^, biomass yield coefficient and maximum specific growth rate^63^, disinfection rate coefficients^64^ and biocide neutralization rate coefficients^65^ were based on typical values from literature. The detachment rate coefficient was selected to produce a steady-state biofilm thickness in the dosing off case of approximately 150 microns. Modifications of these parameters for the cases are in Table 2. The dosing on case (B) has a H_2_O_2_ dosing after 2 days. Case C has the dosing turn on at 2 days and then off after 6 days. Case D is a series of simulations with varying H_2_O_2_ dosing concentrations. Cases E and F explore the influence of the H_2_O_2_ neutralization by the dead and live biomass, respectively. Finally, Case G is a series of simulations that vary the influent glucose concentration.

**Table 1:** Standard parameters used in the simulations.

Parameter	Value	Description
${X}_{t:L}^{0}\,| \,{X}_{t:D}^{0}$	1.0 ∣ 0.0 g/m^3^	Initial concentration of live ∣ dead biomass in the tank
${S}_{t:G}^{0}\,| \,{S}_{t:H}^{0}$	100.0 ∣ 0.0 g/m^3^	Initial concentration of glucose ∣ H_2_O_2_ in the tank
${P}_{b:L}^{0}\,| \,{P}_{b:D}^{0}$	0.08 ∣ 0.0	Initial volume fraction of live ∣ dead biomass in the biofilm
${S}_{b:G}^{0}\,| \,{S}_{b:H}^{0}$	0.0 ∣ 0.0 g/m^3^	Initial concentration of glucose ∣ H_2_O_2_ in the biofilm
${\mu }_{\max }$	9.6 d^−1^	Maximum growth rate of biomass
*K*_*M*_	5.0 g/m^3^	Growth rate half-velocity constant
*Q*	1.0 m^3^/d	Flow rate through tank
*V*	0.1 m^3^	Volume of tank
*A*	1.0 m^2^	Surface area of biomass
*k*_dis_	0.5 (g_*H*_/m^3^)^−1^/d	Disinfection rate
*Y*_*G*,*L*_	0.26 g_*L*_/g_*G*_	Yield coefficient of live biomass on glucose
*S*_in:*G*_	100 g/m^3^	Influent concentration of glucose
dose_1_ ∣ dose_2_	0 ∣ 0 g/m^3^	First ∣ second H_2_O_2_ dosing amount parameter
time_1_ ∣ time_2_	2 ∣ 6 d	First ∣ second H_2_O_2_ dosing time parameter
*k*_*B*:*L*_ = *k*_*B*:*D*_	10 (g_*H*_/m^3^)^−1^/d	Biocide neutralization rate for live and dead biomass
*ρ*_*L*_ ∣ *ρ*_*D*_	2.5e5 ∣ 2.5e5 g/m^3^	Density of live ∣ dead biomass
*D*_*b*:*G*_ ∣ *D*_*e*:*H*_	1.3e-5 ∣ 6.52e-5 m^2^/d	Diffusion coefficient of glucose ∣ H_2_O_2_ in biofilm
*L*_*L*_	1.0e-5 m	Tank boundary layer thickness
*D*_*aq*:*G*_ ∣ *D*_*aq*:*H*_	5.2e-5 ∣ 1.09e-4 m^2^/d	Diffusion coefficient of glucose ∣ H_2_O_2_ in water
${L}_{f}^{0}$	50e-6 m	Initial biofilm thickness
${K}_{\det }$	1e4 m^−1^d^−1^	Biofilm detachment coefficient

**Table 2:** Modification of parameters for various simulation cases.

Case	Description	Modification of standard parameters
A	No Dosing	none
B	Dosing On	dose_1_ = 500 g/m^3^
C	Dosing On/Off	dose_1_ = 500 g/m^3^, dose_2_ = 0 g/m^3^
D	Varying H_2_O_2_ Concentration	dose_1_ ∈ [0, 2*e*4] g/m^3^
E	Only Dead Neutralization	*k*_*B*:*L*_ = 0 (g_*H*_/m^3^)/d
F	Only Live Neutralization	*k*_*B*:*D*_ = 0 (g_*H*_/m^3^)/d
G	Varying Glucose Influent Concentration	*S*_in:*G*_ ∈ [0, 200] g/m^3^

### Reporting summary

Further information on research design is available in the Nature Research Reporting Summary linked to this article.

### Supplementary information

Reporting Summary

## Data Availability

No data is used in this study. The simulated results are produced with Biofilm.jl, which is described in the subsequent section.

## Code availability

The results in this study are produced with the Biofilm.jl software, which is freely available with an MIT license^58^. To run Biofilm.jl with the appropriate inputs and perform postprocessing to generate the figures in this manuscript can be done with a file called HydrogenPeroxideDosing.jl. This file and the version of Biofilm.jl used with this manuscript is available on the HydrogenPeroxidePaper branch of the git repository https://github.com/markowkes/Biofilm.jl/tree/HydrogenPeroxidePaper. A Code Ocean capsule is also provided that reproduces all the figures in this manuscript 10.24433/CO.8353351.v2.
